# Frailty and Quality of Life in Hemodialysis Patients: An Edmonton Frail Scale-Based Assessment in a South Asian Cohort

**DOI:** 10.7759/cureus.110508

**Published:** 2026-06-09

**Authors:** Abdullah Hilal Zafar, Syed Sheheryar Faraz, Sivanaga Venkatalakshmansaivamsidhar Bandi, Ayesha Amal, Anas Javed, Muhammad Asim Fazal, Tahreem Fatima, Sohaib I Uddin, Wania Fatima, Muhammad Ahmad Shahzaib, Uchenna Peter Ohadiugha

**Affiliations:** 1 Accident and Emergency, Royal Albert Edward Infirmary, Wrightington, Wigan and Leigh NHS Foundation Trust, Wigan, GBR; 2 Infectious Diseases, Liaquat National Hospital, Karachi , PAK; 3 Medicine, UV Gullas College of Medicine, Cebu, PHL; 4 General Medicine, Dubai Health, Dubai, ARE; 5 Internal Medicine, Islamic International Medical College Trust (IIMCT)-Railway General Hospital, Rawalpindi, PAK; 6 Nephrology, King Abdullah Medical City, Makkah al-Mukarramah, SAU; 7 Medicine, Rai Medical College, Sargodha, PAK; 8 Acute Medicine, Royal Albert Edward Infirmary, Wrightington, Wigan and Leigh Teaching Hospitals NHS Foundation Trust, Wigan, GBR; 9 Medicine and Surgery, Mohtarma Benazir Bhutto Shaheed Medical College, Mirpur, PAK; 10 Internal Medicine, Henan Medical University, Xinxiang, CHN; 11 Medicine, Afe Babalola University, Abuja , NGA

**Keywords:** edmonton frail scale, frailty, hemodialysis, kdqol, quality of life, south asia

## Abstract

Introduction: Frailty is a complex health condition that can adversely affect both the physical and mental health of patients who are on maintenance hemodialysis. Assessing frailty in this population could help identify patients at risk of adverse outcomes early.

Objective: This study aimed to determine frailty condition through the Edmonton Frail Scale (EFS) and investigate its relationship with kidney disease-related quality of life in South Asian patients receiving hemodialysis.

Methods: A total of 448 adults who had undergone maintenance hemodialysis for at least three months were included in the current cross-sectional study. Frailty was assessed using the EFS, and quality of life was measured using the Kidney Disease Quality of Life-36 (KDQOL-36) questionnaire. The Spearman's rank correlation, the Mann-Whitney U test, the Kruskal-Wallis H test, and multiple linear regression were among the various statistical approaches utilized for data analysis.

Results: Frailty showed a strong inverse relationship with quality of life (ρ = -0.317, p < 0.001). Women had a higher frailty score than men but a lower KDQOL score (p < 0.001). The frailty score was positively correlated with age, while quality of life was negatively correlated with age (p < 0.001). According to regression analysis, greater frailty (B = -0.812), older age, female sex, longer duration of dialysis, more frequent dialysis, and higher comorbidity were significant factors associated with lower quality of life (p ≤ 0.01).

Conclusion: Frailty was significantly associated with reduced quality of life among patients on hemodialysis, highlighting the importance of routine frailty screening during hemodialysis care.

## Introduction

Frailty, a geriatric syndrome, is characterized by reduced physiological reserve and increased susceptibility to adverse outcomes and may be diagnosed using either the physical frailty phenotype or a cumulative deficit index [1, 2]. Several factors, including demographic, lifestyle, and health-related characteristics, contribute to frailty among older adults. Studies show that apart from age, frailty risk is also greater in females, physically inactive individuals, those suffering from other ailments such as diabetes and mental decline, and malnourished individuals [3].

The occurrence of frailty in hemodialysis patients is widespread, and it has a direct relationship with high mortality, hospitalization, and long-term care placement [4]. Among all kidney replacement therapies, hemodialysis is the most common, accounting for nearly 69% of all kidney replacement therapies and approximately 89% of dialysis treatments worldwide [5].

In patients with advanced chronic kidney disease, frailty usually deteriorates during the first year of dialysis, and frailty scores' rise forecasts not only higher hospitalization rates but also mortality [6]. The poor frailty trajectories were associated with higher rates of falls, hospital admissions, and cognitive decline, underscoring the need for early intervention and management of modifiable risk factors [7].

The Edmonton Frail Scale (EFS) is a multidimensional frailty assessment that considers cognition, functional performance, nutrition, mood, social support, and other health-related areas [8]. Understanding frailty status and its association with patient-reported outcomes can improve risk stratification and individualized management in patients receiving maintenance hemodialysis.

This study aims to assess frailty using the EFS and examine its relationship with kidney disease-related quality of life among South Asian patients receiving maintenance hemodialysis. The findings may help support early risk identification and targeted interventions in this vulnerable population.

Study gap 

Frailty has been widely recognized as an important contributor to adverse outcomes among patients receiving hemodialysis. However, most available evidence originates from Western populations, while data from South Asian countries remain limited. Regional differences in socioeconomic conditions, comorbidity burden, healthcare access, and cultural factors may influence the prevalence and impact of frailty in hemodialysis patients. Furthermore, the relationship between frailty and quality of life has not been sufficiently explored in South Asian dialysis populations. A better understanding of frailty and its clinical implications in this setting is essential for early identification of vulnerable patients and the development of targeted interventions to improve patient outcomes.

Objectives of the study

The study aimed to identify the frailty prevalence in the South Asian hemodialysis patients by using the EFS, to investigate the relationship between frailty status and kidney disease-related quality of life among South Asian patients receiving maintenance hemodialysis, and to identify demographic and clinical factors associated with frailty and quality of life among South Asian patients receiving maintenance hemodialysis.

## Materials and methods

Study design

This cross-sectional study evaluated frailty and its relationship with kidney disease-related quality of life among adult patients on maintenance hemodialysis. The cross-sectional design enabled the assessment of frailty prevalence, associated factors, and quality of life at a single time point. The study was conducted from September 25, 2025, to December 6, 2025.

Study population

Adults aged 18 years or older with at least three months of maintenance hemodialysis were eligible to participate in the study. This was the minimum time required to ensure that participants had consistent treatment patterns and were well-exposed to hemodialysis-related physiological stressors.

Patients were omitted when they had a serious medical disease or instability during the time of examination, extreme cognitive impairment or neuropsychiatric conditions such that they would not be able to determine frailty correctly, or failure or refusal to give informed consent.

Sampling technique and sample size

Participants were recruited through convenience sampling from seven dialysis centers located across three cities, including two centers in Sargodha, two centers in Lahore, and three centers in Karachi. Eligible patients were approached during their routine dialysis sessions and invited to participate in the study. Nevertheless, convenience sampling can introduce selection bias because respondents are not randomly selected.

The minimum required sample size was calculated using a 95% confidence level, a 5% margin of error, and an assumed prevalence of 50% in the absence of local prevalence data. Based on these parameters, the estimated minimum sample size was 385 participants. To enhance the precision of the findings and ensure adequate representation of the study population, a total of 448 participants were ultimately recruited and included in the analysis [9].

Data collection

Demographic and Clinical Form

Through a structured form, patient demographics, socio-economic conditions, education, comorbidities (e.g., diabetes, hypertension, cardiovascular diseases), hemodialysis duration/frequency, and lifestyle (physical activity, diet, smoking) were examined. Information regarding comorbidities was obtained through participant self-report during interviews using a structured questionnaire (Appendix A).

Edmonton Frail Scale

The EFS, introduced by Rolfson et al. in 2006, was used to assess frailty [8]. The EFS is a reliable, multifaceted instrument that evaluates nine dimensions, including cognition, general health status, physical independence, social support, drug therapy, nutrition, emotion, bowel control, and physical performance. Scoring on the scale is based on 17 items, which can total from 0 to 17, with higher scores reflecting frailty. Frailty was divided into the following: non-frail (0-4), vulnerable/pre-frail (5-6), mild (7-8), moderate (9-10), and severe (11 and above) [10]. The EFS was administered in its original English, and data were collected through patient interviews. Although the original English version of the EFS was used, questionnaire items were explained verbally in the local language when required to ensure participant comprehension during the interview process.

Kidney Disease Quality of Life (KDQOL-36)

In 1994, Hays and his colleagues introduced KDQOL-36, a tool specifically designed to assess the quality of life of patients with chronic kidney disease. This tool includes 36 items covering several areas, including the Physical Component Summary (PCS), the Mental Component Summary (MCS), the impact of kidney disease, symptoms/problems, and the effects of kidney disease on daily life. The obtained scores range from 0 to 100, with higher scores indicating better quality of life [11]. The original English version was used and distributed through structured interviews during dialysis sessions. Although the original English version of the KDQOL-36 was used, questionnaire items were explained verbally in the local language when necessary to facilitate participant understanding.

Ethical considerations

The study protocol received approval from the Institutional Ethics Committee of Rai Medical College, Sargodha, Pakistan (approval no. RMC/IRB/NEPH/2025/09/012). This approval covered data collection at all participating dialysis centers included in the study. All subjects were notified and provided written consent before data collection. Participant confidentiality was ensured through data anonymization and the secure storage of study records.

Statistical analysis

The data were examined with IBM SPSS Statistics software, version 26.0 (IBM Corp., Armonk, NY, USA). The demographic and clinical characteristics of the 448 hemodialysis participants were summarized using descriptive statistics, including frequencies and percentages. The association between frailty and quality of life was analyzed using Spearman's rank correlation, with frailty quantified by the EFS and quality of life assessed using the KDQOL-36 instrument. Mann-Whitney U tests were used to investigate gender differences in frailty and quality of life. In contrast, Kruskal-Wallis H tests were used to analyze these outcomes across age groups, given that the data were not normally distributed. Finally, the independent factors of quality of life were frailty, age, sex, dialysis characteristics, and comorbidities. The multiple linear regression analysis was performed to obtain these results. All analyses were considered statistically significant with a p-value <0.05. Participants with incomplete questionnaire responses were excluded from the final analysis. No missing data were included in the statistical analyses.

## Results

The study participants were 448, most of whom were older adults, with more than half aged 50 or older. The highest percentage (28.6%) was among those aged 60 and older, as indicated in Table 1. The sample was dominated by females (76.8%), with a majority of the respondents married (55.1%), followed by widowed (27.9%). Education levels were primarily low to moderate, with almost half having primary or secondary education, and only a small percentage having a master's degree or higher (3.6%). In terms of employment, 38.6% were employed, and a very high percentage were unemployed or homemakers. The majority of respondents had a history of receiving hemodialysis for more than one year, especially one to three years (37.1%), and glomerulonephritis (33.7%) and hypertension (25.7%) were the most prevalent causes of kidney disease. Dialysis was carried out most often twice a week (43.1%), and comorbidities were prevalent, particularly cardiovascular disease (35.5%) and hypertension (31.0%), which justifies a significant cardiovascular risk burden among the participants of the study.

**Table 1:** Demographic characteristics of participants (N=448) N=number of participants; f=frequency; %=percentage

Variable	f	%
Age	-	-
18–30 years	40	8.9
31–40 years	70	15.6
41–50 years	90	20.1
51–60 years	120	26.8
>60 years	128	28.6
Gender	-	-
Male	104	23.2
Female	344	76.8
Marital status	-	-
Single	41	9.2
Married	247	55.1
Widowed	125	27.9
Divorced	35	7.8
Educational level	-	-
No formal education	39	8.7
Primary (up to Grade 5)	89	19.9
Secondary (Grade 6–10)	129	28.8
Higher secondary (Grade 11–12)	99	22.1
Bachelor's degree	76	17.0
Master's degree or higher	16	3.6
Employment status	-	-
Student	48	10.7
Employed	173	38.6
Home-maker	91	20.3
Unemployed	113	25.2
Retired	23	5.1
Duration on hemodialysis	-	-
< 6 months	81	18.1
6-12 months	143	31.9
1-3 years	166	37.1
> 3 years	58	12.9
Primary cause of kidney disease	-	-
Diabetes	35	7.8
Hypertension	115	25.7
Glomerulonephritis	151	33.7
Polycystic kidney disease	110	24.6
Unknown	36	8.0
Frequency of dialysis per week	-	-
1 session/week	105	23.4
2 sessions/week	193	43.1
3 sessions/week	150	33.5
Comorbidities	-	-
Diabetes	32	7.1
Hypertension	139	31.0
Cardiovascular disease	159	35.5
Stroke	89	19.9
Chronic lung disease	20	4.5
None	9	2.0

Table 2 displays the results of Spearman's rank-order correlation, which indicates a statistically significant negative correlation between frailty and quality of life (ρ = -0.317, p < 0.001). This means that higher frailty levels, as measured by the Edmonton Frail Scale, are associated with lower quality of life, as assessed by the KDQOL-36.

**Table 2:** Spearman's rank-order correlation between frailty (EFS) and quality of life (KDQOL-36) (N = 448; N = 448) ***=p <0.001 considered significant; positive correlation indicates direct relationship. EFS: Edmonton Frail Scale; KDQOL-36: Kidney Disease Quality of Life-36

Variable	ρ	t(df)	p
The Edmonton Frail Scale (EFS) & Kidney Disease and Quality of Life-36 (KDQOL)	-0.317	-7.06 (446)	<0.001^***^

Table 3 indicates significant differences in frailty and quality of life between male and female hemodialysis patients. Female participants demonstrated significantly higher frailty scores than male participants, as reflected by their higher mean rank on the EFS (312.1 vs. 129.8; Z = -8.15, p < 0.001). Conversely, male participants reported significantly better quality of life than female participants, with higher median KDQOL-36 scores (75 vs. 60) and a lower mean rank for poorer quality-of-life outcomes (Z = -7.76, p < 0.001). These findings suggest that female patients undergoing hemodialysis experience greater frailty and poorer quality of life compared with their male counterparts.

**Table 3:** Gender differences in frailty (EFS) and quality of life (KDQOL-36) among hemodialysis patients (N = 448) N=448 (males=104, 23.2%; females=344, 76.8%); Mann–Whitney U test was used for all comparisons; p-values marked with *** indicate statistical significance at p <0.001***.

Variable	Gender	N	Actual Scores Median (Q1 - Q3)	Mean Rank	Sum of Ranks	Interquartile Range (IQR)	U	Z	p-value
The Edmonton Frail Scale (EFS)	Male	104	10 (7 - 15)	129.8	13,499	4.5	8,039	–	–
	Female	344	12 (9 - 16)	312.1	1,07,362	6	27,737	-8.15	<0.001^***^
Kidney Disease and Quality of Life-36 (KDQOL-36)	Male	104	75 (65 - 85)	138.1	14,362	75	8,902	–	–
	Female	344	60 (50 - 75)	309.2	1,06,365	51	26, 874`	-7.76	<0.001^***^

As shown in Table 4, significant differences in frailty and quality of life were observed across age groups among hemodialysis patients. Frailty increased progressively with age, with participants aged >60 years demonstrating the highest median EFS score (17 (15-17)) and mean rank (264.6), whereas those aged 18-30 years had the lowest median score (6 (4-9)) and mean rank (72.4) (chi square (χ²)(4) = 96.84, p < 0.001). In contrast, quality-of-life scores declined with increasing age, as the youngest age group had the highest KDQOL-36 median score (55 (50-65)) and mean rank (263.2), while participants aged >60 years had the lowest median score (27 (23-33)) and mean rank (74.3) (χ²(4) = 182.17, p < 0.001). These findings indicate that older age is significantly associated with greater frailty and poorer kidney disease-related quality of life among patients receiving maintenance hemodialysis.

**Table 4:** Age-group differences in Frailty (EFS) and quality of life (KDQOL-36) among hemodialysis patients (n = 448) N=number of participants in each age category; %=percentage of the total sample; Percentages are based on total N=448; Values are mean ranks from Kruskal–Wallis H tests; Overall test statistics are reported in the bottom row for each dependent variable: Total EFS (chi-square (χ²)(4) = 96.84, <0.001***) and total KDQOL-36 (χ²(4) = 182.17, <0.001***); Significance levels: p <0.001***.

Variable	Age Group	N	Actual Scores Median (Q1 - Q3)	Mean Rank	Interquartile Range (IQR)	χ^2^(df=4)	p
Edmonton Frail Scale (EFS)	18–30 years	40	6 (4 - 9)	72.4	3.6	–	–
	31–40 years	70	8 (6 - 11)	102.7	4.4	–	–
	41–50 years	90	12 (9 - 14)	157.8	5.6	–	–
	51–60 years	120	15 (12 - 17)	198.5	6.4	–	–
	>60 years	128	17 (15 - 17)	264.6	7.4	96.84	<0.001^***^
Kidney Disease and Quality of Life-36 (KDQOL-36)	18–30 years	40	55 (50 - 65)	263.2	29.4	–	–
	31–40 years	70	50 (45 - 60)	212.7	26.5	–	–
	41–50 years	90	43 (38 - 53)	164.5	23	–	–
	51–60 years	120	36 (30 - 45)	112.8	20	–	–
	>60 years	128	27 (23 - 33)	74.3	16.7	182.17	<0.001^***^

**Figure 1:**
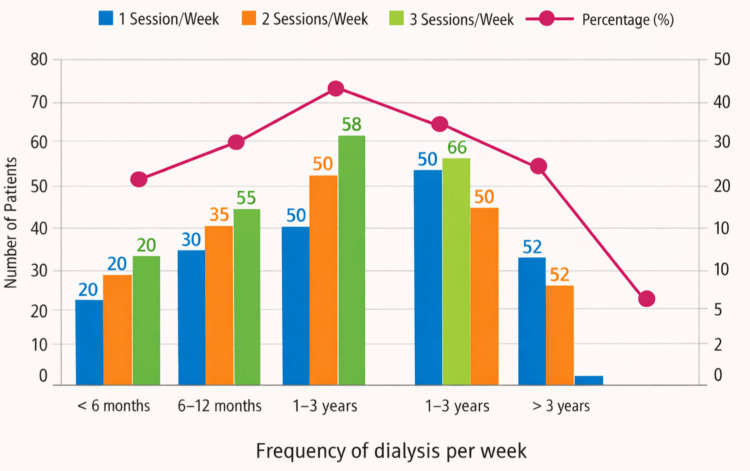
Distribution of frequency of dialysis per week across duration on hemodialysis among patients (N = 448)

Figure 1 illustrates the distribution of dialysis session frequency across different durations of hemodialysis treatment. The highest number of participants was observed in the one to three years duration category. Frequencies of one, two, and three dialysis sessions per week varied across the duration groups and are presented descriptively. No inferential statistical comparisons were performed for this figure.

Multiple linear regression analysis, as shown in Table 5, was performed to examine factors associated with quality of life (KDQOL-36) among hemodialysis patients. The quality of life was significantly associated with higher frailty scores (EFS) (B = -0.812, β = -0.415, p < 0.001). Lower quality of life was also significantly associated with increasing age (B = -3.845, β = -0.433, p = 0.001) and female gender (B = -2.310, β = -0.124, p = 0.001). Besides, longer duration on hemodialysis (β=-0.110, p=0.001), higher frequency of dialysis per week (β=-0.102, p=0.001), and greater comorbidity burden (β=-0.098, p=0.001) were all significantly associated with decreased quality of life. Overall, these findings indicate that frailty, demographic characteristics, dialysis-related factors, and comorbidities are significantly associated with quality of life among hemodialysis patients.

**Table 5:** Multiple linear regression examining factors associated with quality of life (KDQOL-36) among hemodialysis patients (N = 448) Overall model fit (R2 = 0.682; adjusted R2= 0.674; F-statistic = 85.23; overall p-value = <0.001***). Multiple linear regression was conducted to identify factors independently associated with quality of life. Scores; Values include unstandardized coefficients (B), 95% confidence intervals (CI), standard error (SE), standardised beta coefficients (β), and p-values; **=p < 0.01, ***=p <0.001 were considered statistically significant, N = 448. LL: lower limit; UL: upper limit; VIF: variance inflation factor; KDQOL-36: Kidney Disease Quality of Life-36

Predictor	B	SE	β	t	p	95% CI LL	95% CI UL	Tolerance	VIF
(Constant)	60.123	2.870	—	20.95	<0.001^***^	54.504	65.742	—	—
Edmonton Frail Scale (EFS)	-0.812	0.061	-0.415	-13.31	<0.001^***^	-0.932	-0.692	0.83	1.20
Age	-3.845	0.320	-0.433	-12.02	<0.001^***^	-4.473	-3.217	0.72	1.39
Gender	-2.310	0.642	-0.124	-3.60	<0.001^***^	-3.577	-1.043	0.95	1.05
Duration on hemodialysis	-0.912	0.280	-0.110	-3.26	0.001^**^	-1.464	-0.360	0.73	1.37
Frequency of dialysis per week	-1.112	0.322	-0.102	-3.45	0.001^**^	-1.745	-0.479	0.88	1.14
Comorbidities	-0.724	0.215	-0.098	-3.37	0.001^**^	-1.147	-0.301	0.70	1.43

## Discussion

The current research yields valuable information on the burden of frailty and its relation to the quality of life among South Asian patients undergoing maintenance hemodialysis. In this study, frailty was significantly negatively associated with kidney disease-related quality of life, indicating that higher frailty scores were associated with lower kidney disease-related quality of life scores (p < 0.001). These results are consistent with earlier studies reporting that frail CKD patients had significantly lower health-related quality of life scores in both the physical and mental domains compared with non-frail patients across all KDQOL-36 domains [12].

In our study, the frailty scores of female patients were higher, and the kidney disease-related quality of life scores were lower than those of males, which is consistent with earlier studies showing that female gender is an independent risk factor for both frailty and poor health-related quality of life in the hemodialysis population [13,14]. An important finding of this study was the predominance of female participants (76.8%). This distribution differs from many hemodialysis cohorts reported in the literature and may reflect regional differences in healthcare utilization, referral patterns, or sampling characteristics. Therefore, the findings should be interpreted with consideration of the sex distribution of the study population.

Furthermore, age played a key role in influencing both frailty and kidney disease-related quality of life. Older patients had higher frailty scores and lower quality of life scores, confirming systematic reviews reporting that the prevalence and severity of frailty increase with age and that older age is associated with poorer health-related quality of life in dialysis populations [15,16]. Although our findings showed a decline in quality of life with increasing age, not all studies have reported similar results. For example, a previous study reported better mental health-related quality of life among older hemodialysis patients compared with younger patients. Differences in patient characteristics, healthcare systems, cultural factors, and the quality of life domains assessed may partly explain these contrasting findings [17].

The results of our multiple regression analysis indicate that frailty, older age, and female gender were independently associated with lower kidney disease-related quality of life scores, with the strongest independent association being with frailty. This suggests that age, gender, and frailty overlap with patients' quality of life on hemodialysis and align with earlier studies on the same aspect in chronic kidney disease populations [12,14,16].

In terms of treatment factors, longer hemodialysis duration, higher weekly session frequency, and more comorbidities were all associated with slight but statistically significant declines in kidney disease-related quality of life. Previous studies have also shown that prolonged dialysis and increased frequency of sessions are related to slightly worse quality of life, probably because of treatment burden, and patients with comorbidities have lower overall health-related quality of life than those without comorbidities [18-20]. However, not all studies have reported consistent findings. For example, Musavian et al. found no significant relationship between hemodialysis duration and quality of life among patients undergoing dialysis. Differences in patient demographics, healthcare settings, and dialysis practices may partly explain these discrepancies. These findings highlight the complex and multifactorial nature of quality-of-life outcomes among hemodialysis patients [21].

The findings of this study support the incorporation of frailty screening into routine hemodialysis care, particularly among older patients, women, and those with multiple comorbidities. The EFS may serve as a practical screening tool during routine dialysis visits. Early identification of frailty could facilitate targeted interventions, including nutritional counseling, physical rehabilitation, and closer clinical monitoring, to improve quality of life.

Limitations

There are several limitations inherent in this study. First, the cross-sectional design affects causal inference and precludes drawing any conclusions about the temporal relationship between frailty and quality of life. Secondly, convenience sampling may introduce selection bias, thereby limiting the generalizability of the results to the broader hemodialysis population. Thirdly, self-reported measures were used to assess frailty and quality of life, which may be subject to recall and social desirability biases. Moreover, the study lacked both objective physical performance measures and biochemical markers that could have helped to clarify the mechanisms of frailty. Last but not least, although participants were recruited from multiple cities, the findings may not be generalizable to all South Asian hemodialysis populations because the study was conducted within a single country and employed convenience sampling. In addition, the predominance of female participants in the study sample may limit the generalizability of the findings to the broader hemodialysis population. Furthermore, although the original English versions of the EFS and KDQOL-36 were administered with interviewer assistance and verbal explanation in the local language when required, formal linguistic validation of these instruments in the local population was not performed. The distribution of underlying kidney disease etiologies in the study sample may not fully reflect the broader epidemiology of end-stage kidney disease in Pakistan, which may further limit the generalizability of the findings.

Future directions

Longitudinal studies should be the focus of future research to monitor frailty over time and its influence on clinical outcomes, such as hospitalization, falls, and death. The frailty issue should then be addressed through interventional studies evaluating nutritional support, physical rehabilitation, psychosocial counseling, and individualized dialysis strategies to determine whether frailty is reversible and whether targeted interventions can enhance quality of life. Conducting research across several centers and using culture-sensitive frailty assessments may improve the generalizability of the results to the entire population and more clearly reveal differences in frailty patterns among hemodialysis patients from different areas.

## Conclusions

In conclusion, the research reveals that frailty is prevalent among South Asian patients receiving hemodialysis and is strongly associated with quality of life. Frailty was more common among older adults and female patients. Even after adjustment for major demographic and clinical factors, frailty remained independently associated with lower quality of life scores. These findings support the importance of routine frailty assessment and patient-centered care approaches in hemodialysis units. Further longitudinal and interventional studies are needed to determine whether early identification and management of frailty can improve quality of life and clinical outcomes in this vulnerable population.

## Appendices

**Table 6:** Self-reported questionnaire

Items	Responses
Age	-
Gender	-
Marital Status	-
Education Level	-
Employment Status	-
Duration on Hemodialysis	-
Primary Cause of Kidney Disease	-
Frequency of Dialysis per Week	-
Comorbidities (Select all that apply)	-
